# Selenium Silk Nanostructured Films with Antifungal and Antibacterial Activity

**DOI:** 10.1021/acsami.2c21013

**Published:** 2023-02-20

**Authors:** Zenon Toprakcioglu, Elizabeth G. Wiita, Akhila K. Jayaram, Rebecca C. Gregory, Tuomas P. J. Knowles

**Affiliations:** † Yusuf Hamied Department of Chemistry, University of Cambridge, Lensfield Road, Cambridge CB2 1EW, United Kingdom; ‡ Cavendish Laboratory, Department of Physics, University of Cambridge, J J Thomson Avenue, Cambridge CB3 0HE, United Kingdom

**Keywords:** antibacterial and antifungal biomaterials, self-assembly, nanostructured films, selenium nanoparticles, silk fibroin, biocompatible materials, antimicrobial biomaterials

## Abstract

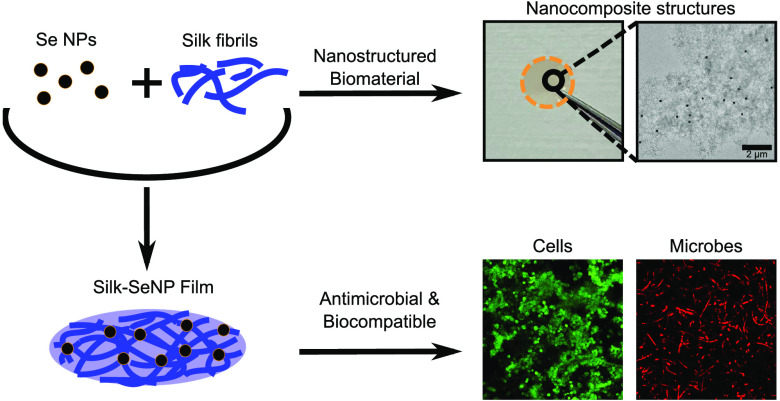

The rapid emergence of drug-resistant bacteria and fungi poses a threat for healthcare worldwide. The development of novel effective small molecule therapeutic strategies in this space has remained challenging. Therefore, one orthogonal approach is to explore biomaterials with physical modes of action that have the potential to generate antimicrobial activity and, in some cases, even prevent antimicrobial resistance. Here, to this effect, we describe an approach for forming silk-based films that contain embedded selenium nanoparticles. We show that these materials exhibit both antibacterial and antifungal properties while crucially also remaining highly biocompatible and noncytotoxic toward mammalian cells. By incorporating the nanoparticles into silk films, the protein scaffold acts in a 2-fold manner; it protects the mammalian cells from the cytotoxic effects of the bare nanoparticles, while also providing a template for bacterial and fungal eradication. A range of hybrid inorganic/organic films were produced and an optimum concentration was found, which allowed for both high bacterial and fungal death while also exhibiting low mammalian cell cytotoxicity. Such films can thus pave the way for next-generation antimicrobial materials for applications such as wound healing and as agents against topical infections, with the added benefit that bacteria and fungi are unlikely to develop antimicrobial resistance to these hybrid materials.

## Introduction

Biomaterials derived from natural sources, including proteins and peptides, provide a unique opportunity to create biocompatible structures for biomedical applications.^1,2^ The self-assembly of these protein molecules can lead to functional complexes that have tunable three-dimensional structures, ranging in size from nanometers to centimeters.^3−7^ Furthermore, these structures offer promising routes for loading, carrying, and releasing cargo molecules to selected targets. More importantly, protein-based structures are nontoxic, nonimmunogenic, biodegradable, and biocompatible, making them ideal candidates for drug/gene delivery applications.^8−11^ A group of materials that distinctly match these applications due to their biobased components are silk-derived proteins.^3,10,12−14^ In its natural role, native silk fibroin (NSF) is spun into long fibers by the *Bombyx mori* silkworm, resulting in strong hydrogen bonding within β-sheet nanocrystallites that have both highly ordered and amorphous regions, giving the silk threads their durable mechanical properties.^15,16^ NSF can be dissolved to form regenerated silk fibroin (RSF),^15,17−19^ resulting in a new material that is a natural block copolymer, which can be readily reassembled into bespoke structures that retain many of the attractive chemical and physical properties that native silk possesses, including durability, biocompatibility, and tunability.^20^ These qualities make it an excellent resource in medical materials, tissue engineering, and in the delivery of therapeutics, with the advantage that RSF can be handled with greater ease than its native counterpart.^12^ Recently, RSF has been used in several medical applications including forming antibacterial materials, wound healing matrices, and targeted drug delivery gels.^3,9,13,21^ Moreover, there has been an increased interest in developing RSF-based structures, which are integrated with nanoparticles, such as selenium nanoparticles, in order to produce antimicrobial materials^22^ that are more prone to killing drug-resistant bacteria and fungi.

Antimicrobial resistance remains one of the most critical public health concerns, as current medicines used to treat a variety of ailments caused by viruses, fungi, and bacteria become ineffective.^23−25^ Research into antimicrobial agents that subsequently do not become resistant to such microbes is thus needed to avert this crisis. Several studies have focused on silver nanoparticles for antimicrobial solutions; however, studies have indicated emerging resistance to these materials from bacterial populations as well as toxicity to human cells.^26−28^ One potential alternative to silver in this role is selenium, a trace element in the human body that is an essential micronutrient often found as selenocysteine, the 21st human amino acid.^29,30^ Selenium nanoparticles have been shown to exhibit effective antimicrobial properties^31−33^ against a broad range of microorganisms including *Escherichia coli* and *Candida albicans*.^32^ Moreover, there has been an increased interest in functionalizing selenium nanoparticles using chitosan,^34^ hyaluronic acid,^35^ polyethylene glycol (PEG),^36^ pectin,^37^ and peptides,^38^ in order to improve stability and enhance biocompatibility.^39^ Selenium’s naturally occurring presence in the human body makes it an excellent alternative for antimicrobial development, because it is much less toxic to humans than silver and is unlikely to develop antimicrobial resistance.^40−42^ Importantly, the antimicrobial properties and efficacy of selenium nanoparticles depend on their size and arrangement within a material to effectively inhibit microbial growth.

In this study, we have developed hybrid selenium-silk based films capable of potent antimicrobial properties and with excellent biocompatibility. In order to do this, we first optimized the formation of selenium nanoparticles (SeNPs) through the reduction of sodium selenite by ascorbic acid.^43^ By varying the concentration of sodium selenite from 100 μg/m to 1000 μg/mL, we were able to form nanoparticles of different sizes. Using electron microscopy, we determined the size distribution of the nanoparticles over a 6-day period. We found that nanoparticle diameters varied (60–130 nm) as a function of the sodium selenite concentration. Additionally, the stability over time was monitored and it was found that the nanoparticles agglomerated as time progressed, with complete agglomeration happening by day 6. This issue was addressed by combining the nanoparticles with silk fibrils, which acted as stabilizers and did not allow the nanoparticles to clump together. These hybrid silk–selenium films demonstrate unique malleability and have a homogeneous nanoparticle distribution within the protein matrix. Finally, we looked at the potential use of these films as antimicrobial agents. Three different bacteria and fungi were investigated: the Gram-negative bacterium *E. coli*, the Gram-positive bacterium *Bacillus subtilis*, and the fungi *Candida parapsilosis*. In all systems, it was determined that the films displayed potent antifungal and antibacterial properties, killing the majority of the bacteria/fungi. More importantly, however, we found that, by incorporating the SeNPs within the films, mammalian cells were protected against the cytotoxic effects of the bare nanoparticles. Therefore, we were able to not only retain the antimicrobial effect of the nanoparticles, but also ensure that our material was highly biocompatible, making these silk–selenium films ideal for use as topical antimicrobial agents.

## Materials and Methods

### Formation and Purification of Regenerated Silk Fibroin

Regenerated silk fibroin was obtained from *Bombyx mori* silkworm cocoons, following a previously determined protocol.^16^ In summary, cut cocoons were boiled for 30 min in a solution of 0.02 M sodium carbonate. The remaining silk product was rinsed with cold water and dried overnight. A 1:4 ratio of 9.3 M lithium bromide solution was added to the silk fibroin and heated at 60 °C for 4 h. The salt was then removed using dialysis and the product was incubated for 2 days with several water changes. The resulting silk fibroin solution was purified by centrifugation and stored at 4 °C.

### Synthesis of Selenium Nanoparticles

SeNPs were prepared by optimizing a previously established protocol in which sodium selenite is reduced by ascorbic acid.^43^ Briefly, five samples of 1800 μL Na_2_O_3_Se in Milli-Q water were prepared (100, 200, 330, 450, 660, 800, and 1000 μg/mL). Ascorbic acid (200 μL, 56.7 mM) was added dropwise to the sodium selenite solution and was mixed thoroughly to obtain the SeNPs. A color change occurred upon nanoparticle formation from translucent colorless to translucent red.

### Characterization of Selenium Nanoparticles

Transmission electron microscopy (TEM), scanning electron microscopy (SEM), ultraviolet–visible (UV-vis) absorption spectroscopy, dynamic light scattering (DLS), and powder X-ray diffraction (PXRD) were performed to characterize our products. The size and distribution of the SeNPs were studied by TEM. TEM micrographs were recorded using a Thermo Scientific (FEI) Talos F200X G2 electron microscope. Samples were prepared over a period of 6 days in which nanoparticles were drop-casted on carbon-coated TEM copper grids and dried after 30 s of exposure. The resulting TEM micrographs were analyzed using Siemens Totally Integrated Automation (TIA) software and ImageJ software.

SEM analysis was employed to image the nanoparticles using a FEI Verios 460 SEM at a current of 5 kV. The SeNPs solution was mounted onto a silicon wafer by adding 1 μL of solution and letting the sample air-dry. This wafer was placed onto a multipin specimen holder, and a 10 nm platinum layer was sputter-coated onto the sample.

SeNPs were also characterized by UV-vis absorption spectroscopy (Implen NanoPhotometer NP80). The samples were diluted by a factor of 100, and the spectra were recorded within a wavelength range of 200–400 nm over a period of 6 days. The average particle size (hydrodynamic diameter) of the synthesized nanoparticles was measured using DLS. The samples were diluted by a factor of 1000, and the spectra were recorded on a Malvern Zetasizer Nano instrument. Nanoparticle composition was characterized using a Bruker D8-QUEST PHOTON-100 diffractometer. The resulting crystallite size was compared to the standard crystalline selenium diffraction pattern.

### Incorporation of Selenium Nanoparticles into Silk Fibroin Films

A solution of regenerated silk fibroin was mixed in a 1:1 ratio with ethanol. This, in turn, was mixed with a 1:1 ratio with each concentration of SeNPs, yielding final nanoparticle concentrations of 50, 100, 165, 225, 300, 400, and 500 μg/mL. The final solutions were pipetted onto a weighing boat and left to dry overnight, resulting in the formation of a film. The film was peeled off and cut using a hole puncher. This was put into a 96-well plate in order to conduct the antimicrobial assays.

### PXRD Analysis

PXRD analysis of the 660 μg/mL SeNP sample was performed using a Panalytical Empyrean diffractometer. The sample was added dropwise in five applications to a single-crystal silicon sample holder and dried at 50 °C between applications. The sample was measured for 15 h. The Se peaks in the spectra match the expected reference peaks indicated by the red lines.

### Transmission Electron Microscopy (TEM) and Energy-Dispersive X-ray (EDX) Spectroscopy Analysis

TEM, high angle annular dark field scanning transmission electron microscopy (HAADF-STEM), and energy-dispersive X-ray (EDX) spectroscopy were performed using a Thermo Scientific (FEI) Talos F200X G2 TEM system that was operating at 200 kV. TEM images were acquired using a Ceta 16 M CMOS camera. EDX spectra were collected using the Super-X EDS detector system, which consisted of four windowless silicon drift detectors. The peak positions in Figure S3 in the Supporting Information (SI) (shown in red) recorded at 2θ = 23.9°, 30.0°, 41.7°, 44.0°, 45.7°, 52.0°, 56.4°, 62.2°, 65.5°, and 68.4° are characteristic of the (100), (101), (110), (102), (111), (201), (003), (202), (210), and (211), respectively, and are representative of a pure hexagonal phase of selenium crystals with lattice parameters *a* = 4.366 Å and *c* = 4.9536 Å^44^ (see Figure S3). TEM grids (continuous carbon film on 300 mesh Cu) were glow-discharged using a Quorum Technologies GloQube instrument at a current of 25 mA for 60 s. Samples were either observed as prepared or negatively stained using a 2% uranyl acetate solution for 45 s.

### Kinetic Growth Analysis

*E. coli* bacterial cells were grown to an optical density measured at a wavelength of 600 nm (OD600) of 0.2 in LB media. Film samples were placed within the wells of a 96-well plate and bacterial solutions were added on top. Kinetic growth inhibition was determined by turbidity analysis via optical density measurements (at 600 nm), using a FLUOstar Omega microplate reader (BMG Labtech). Three independent experiments were conducted, and the results presented are indicative of the triplicates.

### Viability Analysis of Bacterial and Fungal Cells

*E. coli* and *C. parapsilosis* bacteria were incubated at 37 °C with films containing nanoparticles of various concentrations in well chambers. Similarly, *C. parapsilosis* fungi was incubated at 30 °C with films containing nanoparticles in the same format. Post-incubation, bacteria were extracted using a pipet and transferred to an Eppendorf tube. Syto 9 and propidium iodide (LIVE/DEAD BacLight Bacterial Viability Kit, Thermo Fisher Scientific, TFS) were subsequently added in a 1:1 ratio following a 10× dilution. The bacterial cells were immediately observed under a 40× oil objective, using a Leica TCS SP8 inverted confocal microscope.

### Confocal Microscopy and Cell Membrane Study

Confocal microscopy was employed to study the mechanism behind bacteria eradication. SYTOX Blue (Thermo Fisher Scientific) 1 μM was incubated with *E. coli* at an OD600 of 0.1 for 30 min at 37 °C. The sample was then examined using confocal microscopy (Zeiss, Model LSM 510), excited at 405 nm (Leica, Model TCS SP8).

### Cell Culture of HEK-293 Cells

Human embryonic kidney 293 (HEK-293) cells were cultured in 25 cm^2^ flasks at 37 °C and 5% CO_2_ using advanced Dulbecco’s modified Eagle medium (DMEM; TFS) with the addition of 10% fetal bovine serum (Merck), 50 U/mL penicillin and 50 μg/mL streptomycin (TFS), 1% (v/v) GlutaMax (TFS), and 50 μg/mL gentamicin (TFS).

### Cytotoxicity and Cell Proliferation Using MTT Assay on HEK-293 Cells

The viability of HEK-293 cells following incubation with films was determined using a standard MTT cell proliferation assay. Approximately 10^5^ cells were seeded per well in a 96-well plate and incubated for 24 h under 37 °C and 5% CO_2_. Following this, 10 μL of MTT (3-[4,5-dimethylthiazol-2-yl]-2,5-diphenyltetrazolium bromide) labeling reagent, Merck) was added to each well with an incubation period of 4 h. Post-incubation, 100 μL of the solubilization solution was added to each well and a further overnight incubation was performed at 37 °C and 5% CO_2_. The absorbance of the resulting purple solution was measured at 595 nm using a FLUOstar Omega microplate reader (BMG Labtech).

### Viability Analysis of HEK-293 Cells

The viability of HEK-293 cells post-exposure to films was also determined using a LIVE/DEAD Viability/Cytotoxicity Kit (Invitrogen). Approximately 10^5^ cells were seeded per well, in which films were predeposited. The cells were incubated for 24 h, and a stock solution containing 5 μL calcein AM (Component A) and 20 μL ethidium homodimer-1 (Component B) was added to 10 mL Dulbecco’s PBS to create a stock solution. 100–200 μL of the stock dye solution was added to each well and the cells were observed under 10× and 40× objectives in a Leica Model TCS SP8 inverted confocal microscope to determine the relative number of live and dead cells.

## Results and Discussion

**Figure 1:**
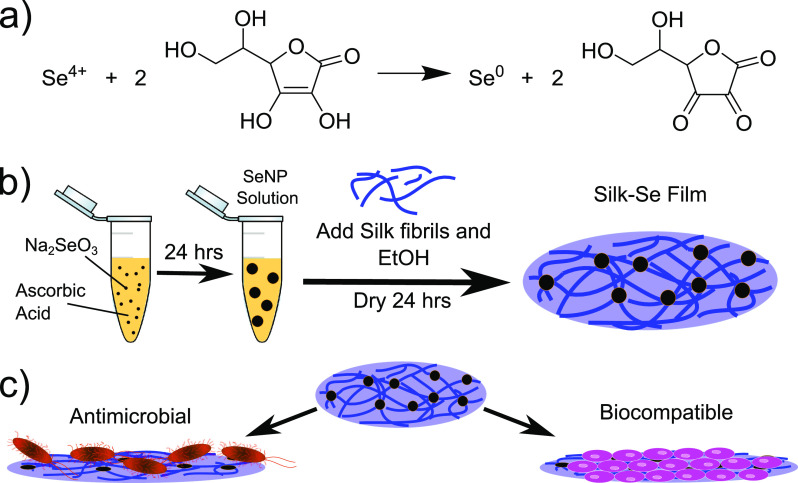
(a) Redox reaction for the synthesis of SeNPs. Selenium is reduced by ascorbic acid. (b) Schematic representation of the experimental procedure used to make the SeNPs. The resulting nanoparticles are combined with silk fibrils and EtOH to form a silk–selenium film. (c) The silk–selenium films were then used as antimicrobial agents while also presenting a high degree of biocompatibility.

In order to generate a material that displays antimicrobial properties while also being biocompatible, we chose to investigate a hybrid inorganic/organic system. Since selenium has been shown to exhibit antimicrobial properties, SeNPs were synthesized using a redox reaction (Figure 1a). During this process, selenium is reduced from a 4+ oxidation state to 0. The SeNPs were then integrated with silk fibrils, before being left to self-assemble, resulting in the formation of a silk-SeNP film (Figure 1b). The films were then tested with fungi, as well as Gram-negative and Gram-positive bacteria, and it was found that, in all cases, microbial death was observed. Finally, viability assays were conducted with mammalian cells and it was determined that crucially, the films remain highly biocompatible (Figure 1c).

As described above, selenium nanoparticles (SeNPs) were first synthesized. A range of sodium selenite concentrations were investigated, while the ascorbic acid solution, which was in excess with respect to the sodium selenite, was kept constant at a concentration of 56.7 mM. Following the reduction reaction, the solution was left at room temperature for 24 h in order to promote SeNP formation. Selenium nanoparticles were imaged by transmission electron microscopy (TEM) and scanning electron microscopy (SEM). From the TEM micrographs, it was determined that the average sizes of the nanoparticles for the 7 different concentrations of sodium selenite investigated, ranged from around 60 to 130 nm. Moreover, depending on the concentration of sodium selenite, we could systematically control the nanoparticle size. The largest SeNPs were formed using a sodium selenite concentration of 200 μg/mL.

**Figure 2:**
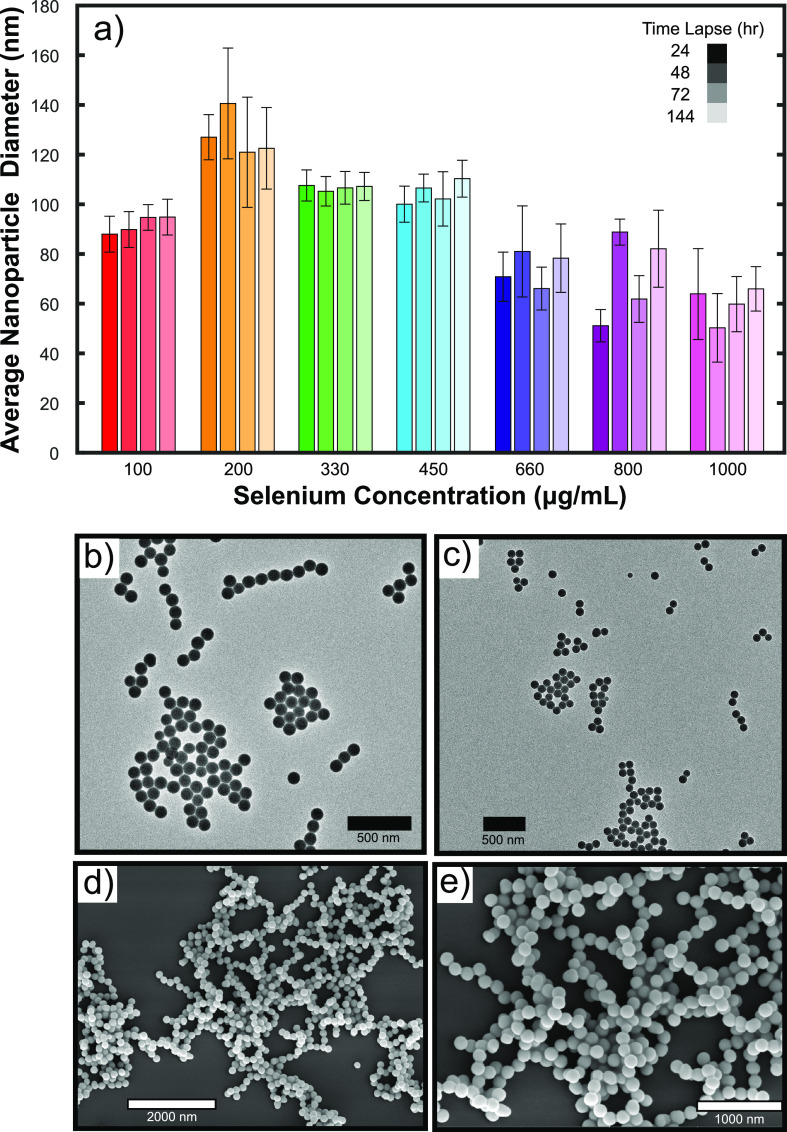
(a) Average diameter of SeNPs at 24, 48, 72, and 144 h as a function of SeNP concentration for seven samples: (red) 100 μg/mL, (orange) 200 μg/mL, (green) 330 μg/mL, (light blue) 450 μg/mL, (dark blue) 660 μg/mL, (purple) 800 μg/mL, and (pink) 1000 μg/mL. TEM micrographs for two samples: (b) 200 μg/mL and (c) 800 μg/mL. (d, e) SEM micrographs of SeNPs at a concentration of 200 μg/mL.

For each sodium selenite concentration used, the size of more than 100 nanoparticles was measured. The mean size (μ) and the standard deviation (σ) of the nanoparticles was thus determined. The average coefficient of variation (which is the ratio of σ to μ and gives an indication of the dispersion of the distribution) for these samples was calculated as 9.8%, demonstrating a fairly narrow distribution for most of the nanoparticle systems synthesized. The average sizes of the SeNPs for the seven conditions tested can be seen in Figure 2a, while some representative TEM and SEM micrographs of the nanoparticles can be seen in Figures 2b and 2c and in Figures 2a and 2e, respectively.

Next, we explored the stability of the SeNPs over time. It is known that, with time, nanoparticles have the tendency to degrade and form aggregated structures, resulting in changes in their size distribution. In order to monitor this process, we looked at the size distribution of the SeNPs for all seven systems over a period of 144 h. TEM was employed to monitor the time-dependent size distribution and, again, more than 100 nanoparticles were measured for each bar graph, as shown in Figure 2a. It was determined that, for all the systems, SeNPs remained stable over time with minimal size discrepancies. The different colors in Figure 2a represent the different nanoparticle concentrations, while the color gradient corresponds to the day on which the measurements were made. It is clear that the size distribution of the SeNPs follows the same trend in day 2 as in day 6.

Moreover, in order to probe the elemental composition of the nanoparticles, energy-dispersive X-ray spectroscopy (EDX) was performed on a nanoparticle sample. As can be seen in Figure S2 in the SI, the Se peaks at 1.5 keV confirm that the nanoparticles imaged were, in fact, selenium-based, with other peaks characteristic of the copper grid used for sample deposition and of the staining agent, uranyl acetate. Furthermore, powder X-ray diffraction (PXRD) was conducted on a 600 μg/mL nanoparticle solution. The peak positions (shown in red) are representative of a pure hexagonal phase of selenium crystals with lattice parameters of *a* = 4.366 Å and *c* = 4.9536 Å^44^ (recall Figure S3).

**Figure 3:**
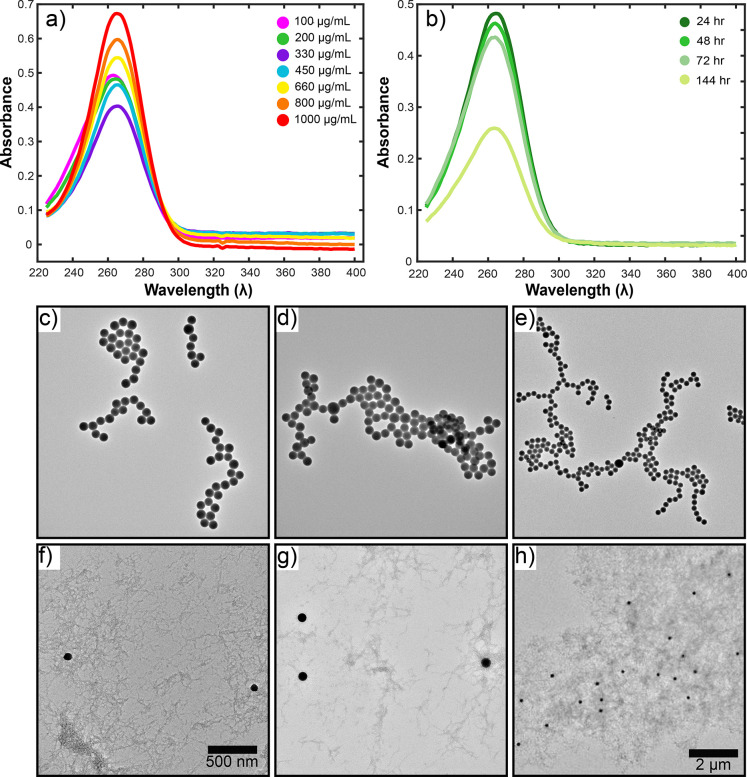
(a) UV-vis absorption spectra for SeNPs taken at day 2. Seven different samples were tested: (pink) 100 μg/mL, (green) 200 μg/mL, (purple) 330 μg/mL, (blue) 450 μg/mL, (yellow) 660 μg/mL, (orange) 800 μg/mL, and (red) 1000 μg/mL. (b) UV-vis absorption spectra for a 200 μg/mL nanoparticle solution at 24, 48, 72, and 144 h. (c–e) TEM micrographs of SeNPs without silk fibrils and (f–h) with silk fibrils. Silk fibrils prevent the nanoparticles from agglomerating.

To monitor the stability of the nanoparticles, the use of UV-vis absorption spectroscopy was employed. Absorption spectra of SeNPs were taken and are depicted in Figures 3a and 3b. The absorption intensity of the nanoparticles at their characteristic wavelength (265 nm) is an indication of the stability, where the higher the intensity, the more stable the nanoparticles.^43^ From the absorption spectra in Figure 3a, which correspond to the different sodium selenite concentrations tested, it can be seen that the most stable system is at 1000 μg/mL, while the least stable nanoparticle system is at 330 μg/mL. Moreover, by monitoring an intermediate stability nanoparticle system over time, at 200 μg/mL, it was determined that there is a clear decrease in the absorbance at 265 nm (Figure 3b), which indicates that the nanoparticles start to clump and aggregate over time.

It was determined that the nanoparticles aggregated for all concentrations tested. Moreover, even though this agglomeration process was initiated from day 3 onward, complete agglomeration occurred by day 6, as can be seen in the TEM micrographs in Figures 3c–e. This was also corroborated from the absorbance spectra at 265 nm (Figure 3b), where it is clear that the intensity of the nanoparticle spectra decreases over time. Furthermore, massive clumps, on the order of millimeters, were seen after 2 weeks of formation (see Figure S1 in the SI). In order to address the issue of nanoparticle agglomeration, regenerated silk fibroin (RSF) was used. RSF is an FDA approved protein, which has the propensity to self-assemble into nanofibrils. Silk protein’s propensity to self-assemble and form fibrillar networks makes it an ideal candidate for use in biomaterials. The formation process is thermodynamically and kinetically favorable due to the presence of repeating hexapeptides GAGAGS and GAGAGY. These sequences form hydrophobic groups in the chain and promote the assembly of β sheet structures.^45,46^ By combining SeNPs with silk nanofibrils, we were thus able to stabilize the nanoparticles and prevent agglomeration. The nanoparticles had the tendency to adsorb to the fibrillar network and were therefore spatially immobilized. This mechanism of stabilization was confirmed via TEM, and some typical micrographs can be seen in Figures 3f–h. The resulting product is a silk-based film with SeNPs dispersed throughout the structure.

Having established that silk fibrils stabilize SeNPs against aggregation, we next sought to form a material capable of exhibiting antimicrobial properties. The silk fibril–SeNP (SF-SeNP) hybrid inorganic/organic solution was mixed with a 40% ethanol solution (which is a strong gel promoter) at a 1:1 ratio, and the final mixture was left to dry at room temperature for 1 day. The experimental procedure used to make the films is shown in the schematics in Figures 1a and 1b. This process resulted in the formation of a SF-SeNP film (as can be seen in schematically Figure 1b and in Figures S4b and S4c in the SI). By allowing the solution to dry at room temperature, a film with a uniform nanoparticle distribution could be formed. In order to monitor the antimicrobial properties of the films, we prepared SF-SeNP films for the seven different concentrations tested, i.e., the concentration range of 50–500 μg/mL. The films were then cut using a hole puncher and placed in a 96-well plate, in order to conduct absorbance- and fluorescence-related assays using a plate-reader experiment.

**Figure 4:**
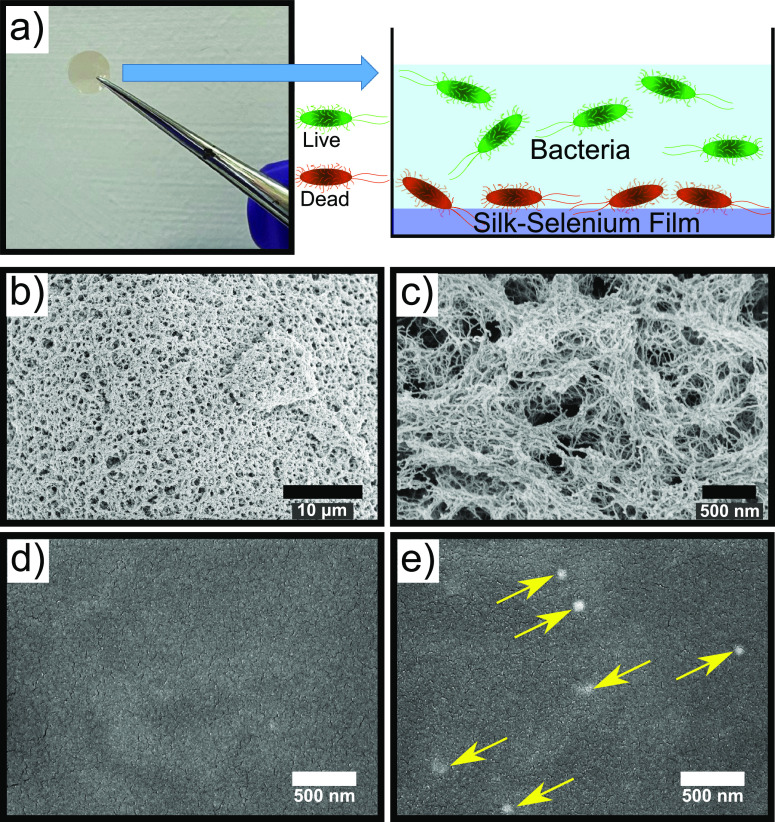
(a) Schematic representation of the experimental procedure used to incubate the silk–selenium films with the microbes. (b, c) High-magnification SEM micrographs of silk films showing the highly dense fibrillar network. (d, e) SEM micrographs of silk films in the absence of SeNPs (panel (d)) and the presence of SeNPs ((panel (e)). The yellow arrows point toward the nanoparticles within the film.

Before investigating the antimicrobial properties of the SF-SeNP films, we first characterized the films using SEM analysis. Micrographs of both silk films with and without SeNPs were taken. High-magnification SEM micrographs revealed the highly dense fibrillar network with the silk films (Figures 4b and 4c). A representative image of a silk film without nanoparticles can be seen in Figure 4d. It contains a homogeneous distribution of the self-assembled protein network. Moreover, in the micrographs of the SF-SeNP films (Figure 4e), the nanoparticles can clearly be seen, with yellow arrows pointing toward the SeNPs within the film.

**Figure 5:**
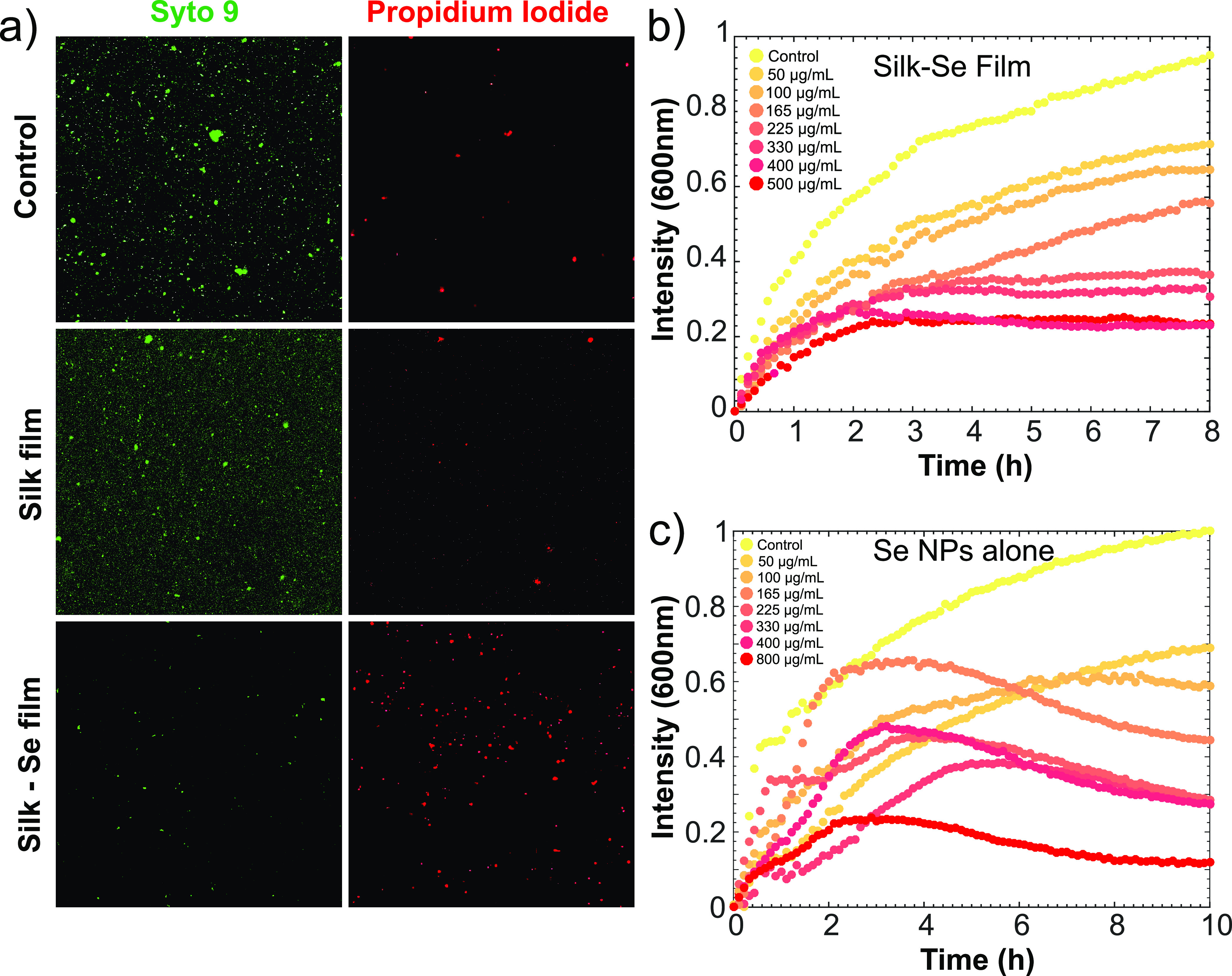
(a) Bacterial viability analysis of *E. coli* via live/dead staining following overnight treatment with and without silk–selenium films. A 40× magnification was used for all micrographs. (b) Kinetics of bacterial growth inhibition on a silk–selenium film via turbidity analysis. (c) Kinetics of bacterial growth inhibition using bare selenium nanoparticles obtained via turbidity analysis. The absorbance measurements were done at 600 nm.

Following film formation, we then evaluated the antibacterial activity of the SF-SeNP films against *E. coli*. Films were first deposited into a 96-well plate before antimicrobial assays were conducted. Direct assessment of bacterial viability of *E. coli* was carried out using live/dead viability analysis. This was achieved through use of Syto9 (a dye that stains and indicates live bacteria) and propidium iodide (a dye that stains and indicates dead bacteria). Following 24 h of treatment, bacterial cell death was observed for samples treated with the SF-SeNP films (bottom panel of Figure 5a). This effect was observed for a range of nanoparticle concentrations, with the 500 μg/mL system showing the highest potency. Furthermore, the control and silk film treated bacteria remained viable and proliferated over time, as shown in Figure 5a (top and middle panels, respectively).

Moreover, the effect of selenium nanoparticle concentration on bacterial cell death was investigated. This was done by conducting a kinetic growth inhibition analysis. *E. coli* cultures, grown to an OD of 0.2, were added to the nanoparticle containing films and to the corresponding controls. All experiments were conducted under constant shaking at 250 rpm until measurements were performed. This was done in order to ensure that bacterial precipitation did not occur. The bacteria were incubated overnight and absorbance measurements at 600 nm were conducted over time. It is evident from Figure 5b that there is a dose-dependent trend, with the higher the nanoparticle concentration, the more potent the antibacterial properties of the film. Treatment with the silk films alone did not affect bacterial growth (Figure 5b). Additionally, it was observed that the SF-SeNP films with concentrations of 225 μg/mL and above had >70% bacterial death. It is important to note that we did not see complete eradication of *E. coli*, but rather bacterial inhibition was observed. We also performed a colony growth on agar plates in the presence and absence of SF-SeNP films. Images of agar plates with and without films clearly show that, in the absence of films, bacteria grow (top 2 panels in Figure S5a in the SI), whereas, in the presence of films, bacterial growth is inhibited (bottom two panels in Figure S5b in the SI). The drawn in black area represents the outline of the colonies on day 1, whereas the red circle represents the outline of the films once placed over the colonies.

In order to assess whether the SF-SeNP films retain their antibacterial potency, as opposed to using just bare nanoparticles, the kinetic inhibition analysis was also performed for free nanoparticles in solution. The bacteria were again incubated overnight and absorbance measurements at 600 nm were conducted over time. It is evident from Figure 5c that a similar dose-dependent trend is observed, as in the case of the SF-SeNP films. It was found that with bare nanoparticles, a similar amount of bacteria were killed (∼70% bacterial death) for nanoparticle concentrations up to 400 μg/mL. Therefore, we can conclude that our SF-SeNP films retain their antibacterial action and have similar performances to using free nanoparticles.

**Figure 6:**
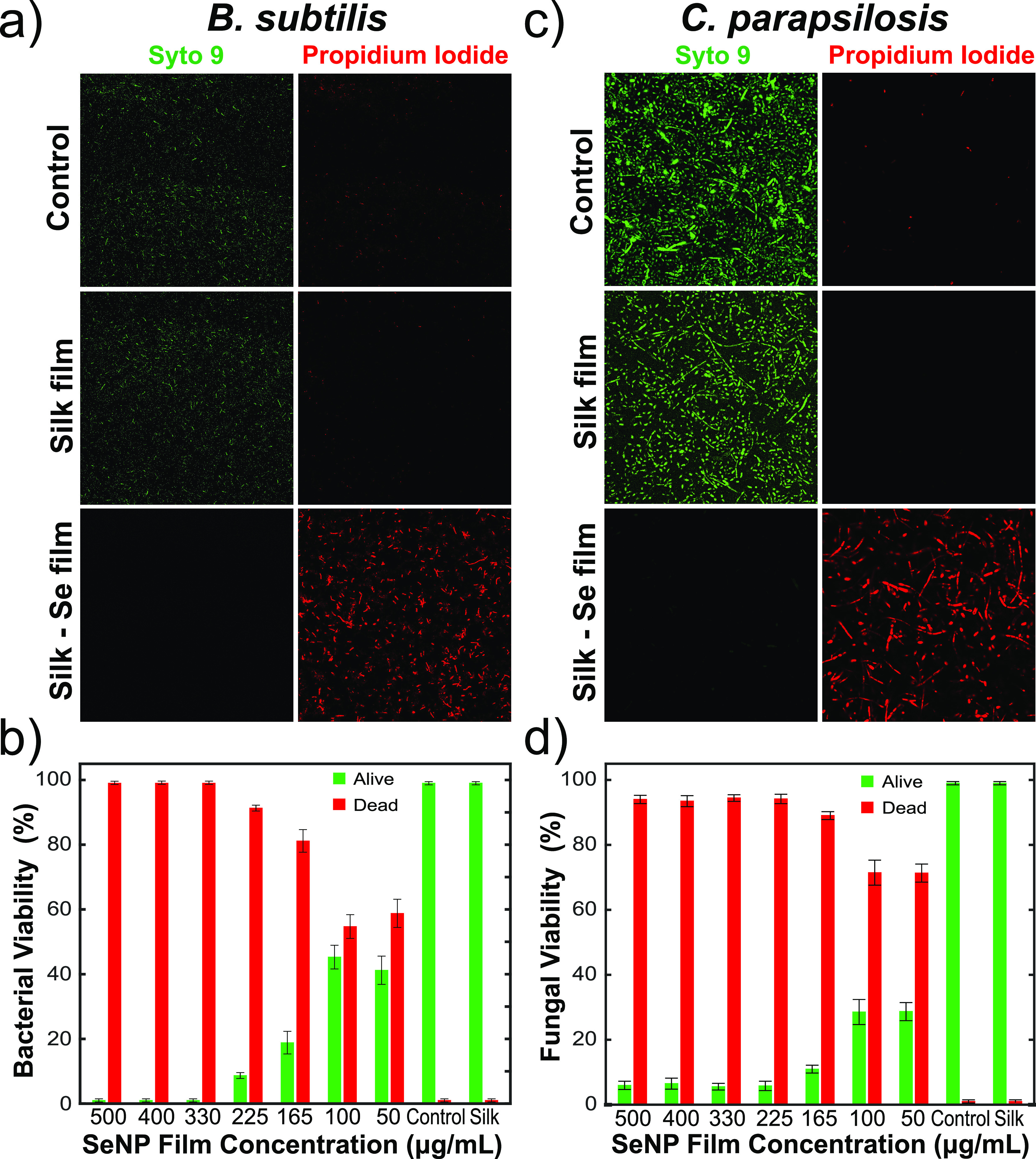
(a) Bacterial viability analysis of *B. subtilis* via live/dead staining following overnight treatment. A 40× magnification was used for all micrographs. (b) Quantification of bacterial viability of *B. subtilis* on selenium films. (c) Fungal viability analysis of *C. parapsilosis* via live/dead staining following overnight treatment. A 40× magnification was used for all micrographs. (d) Quantification of fungal viability of *C. parapsilosis* on selenium films. For each condition investigated in panels (b) and (d), 10 different repeats were conducted and *n* > 330 cells were measured for each condition.

Furthermore, the antibacterial activity of the films on a Gram-positive bacterium, *B. subtilis*, was investigated. It has been shown that, due to the selenium nanoparticles having an overall negative charge, they are more potent on Gram-positive bacteria.^47^ Therefore, it was expected that, for the same nanoparticle concentrations, the antibacterial efficacy of the films would be greater on *B. subtilis* than on the Gram-negative bacterium *E. coli*. Bacterial viability was again assessed after 24 h of treatment using Syto9 and propidium iodide, the results of which are summarized in Figure 6a).

We next quantified the viability of the *B. subtilis*, as a function of nanoparticle concentration within the films, as shown in Figure 6b. This was done by counting the live/dead cells from the confocal images taken. For each condition investigated, 10 different repeats were conducted. Moreover, *n* > 330 cells were measured for each condition. Our results indicate that at a concentration of 300 μg/mL and above, total bacterial eradication was observed, whereas, at a concentration of 225 μg/mL, more than 92% of *B. subtilis* was killed. Importantly, even at really low nanoparticle concentrations (50 μg/mL), ca. 60% bacterial death was observed.

Moreover, the antifungal activity of the films was investigated on the *Candida parapsilosis* fungi. Previous literature has shown that SeNPs have good antifungal activity.^40^ We found that our hybrid films had a very potent antifungal effect. Fungal viability was assessed using a similar approach as mentioned above, with the results summarized in Figure 6c). We quantified the effect of the films on *C. parapsilosis* by counting the live/dead cells from the confocal images, as previously mentioned. The antifungal effect of the SF-SeNP films (Figure 6d) is more potent than their antibacterial effect, with ∼73% fungal death observed for the films with nanoparticle concentrations as low as 50 μg/mL. More importantly, the concentration that kills the majority of fungi (>95%) is 225 μg/mL and above.

In order to gain a mechanistic insight into the mode of action of the SF-SeNP films, a membrane permeation assay was conducted with SYTOX Blue. SYTOX Blue is a cationic dye that cannot enter an intact cell, but rather it can only enter if the membrane has been disrupted. The dye binds to intracellular nucleic acids and is fluorescent when excited at 405 nm.^21^ Samples treated with SF-SeNP films had significantly higher fluorescence signal, compared to control samples, indicating that membrane disruption was evident, and, thus, is the probable mode of action behind the antimicrobial effect of the SF-SeNP films (Figure S6 in the SI).

The mechanism by which SeNPs eradicate bacteria/fungi is via generation of reactive oxygen species (ROS). These are known to bind intracellular components, thereby disrupting biological membranes.^48−51^ These species are particularly efficacious in a biological setting as they can damage essential biomolecules such as DNA and lipids. Furthermore, ROS can inhibit ATP production and DNA replication, which prevents the cells’ built-in antioxidant defense system from working and can lead to cell damage or cell death.^52,53^ In the context of selenium, SeNPs have been known to kill bacteria due to the presence of ROS, which, as mentioned above, disrupts the cell membrane and results in bacterial cell death.^40,50,54−56^ As the antimicrobial activity in our system originates from membrane disruption due to ROS (Figure S6), bacteria and fungi are therefore highly unlikely to develop a resistance to our hybrid biomaterials.

**Figure 7:**
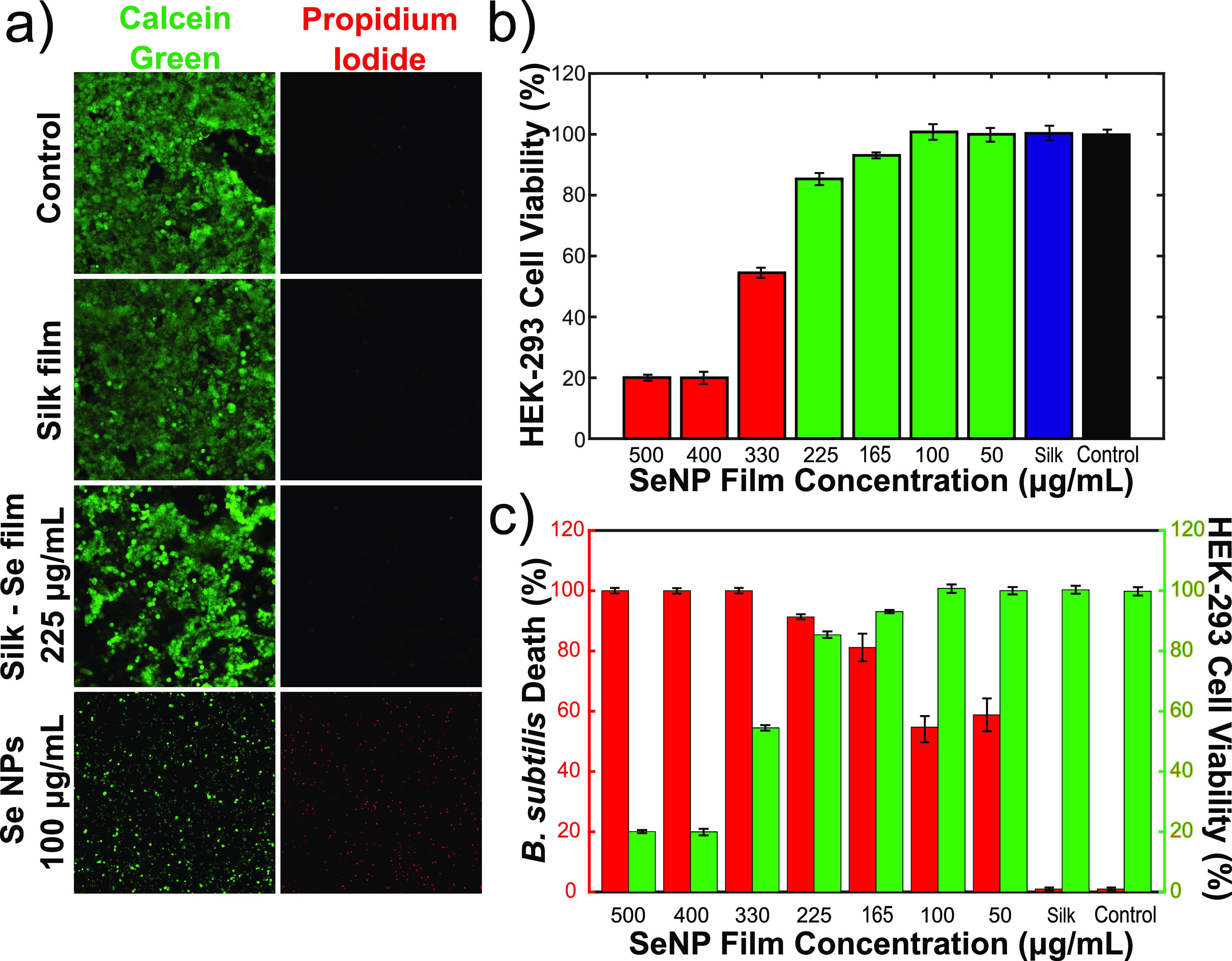
(a) Mammalian cell viability analysis with the antibacterial films and controls following an overnight incubation. This was conducted using a fluorescence-based live/dead staining assay containing fluorescein diacetate (live cells) and propidium iodide (dead cells). A 40× magnification was used for all the microscopy images. (b) MTT cell viability analysis of HEK-293 cells with the antibacterial films and controls. (c) Combined graph of the antibacterial activity on *B. subtilis* and HEK-293 cell viability, as a function of the silk–selenium film concentration.

Finally, in order to assess the ability of these films as agents for treating topical infections, the biocompatibility of the antibacterial films with mammalian cells (HEK-293) via an MTT-based cell viability assay was evaluated. HEK-293 cells were grown in 96-well plates overnight in the presence of the films and controls. Cell viability was not affected by the presence of a silk film. Moreover, in the presence of films containing low nanoparticle concentrations (100 μg/mL and below), high cellular viability was observed (Figure 7b). Cellular viability was reduced to just over 85% when treated with films containing a SeNP concentration of 225 μg/mL. However, the viability was reduced to ∼20% when the cells were treated with films containing 400 and 500 μg/mL (Figure 7b).

Additionally, a live/dead analysis of HEK-293 cells which were treated in a similar manner indicated the same results. Calcein AM staining (indicating live cells) and ethidium homodimer-1 (indicating dead cells) was conducted (Figure 7a). From the images, it is clear that there is minimal cell death in the presence of films, both with and without nanoparticles (top three panels of Figure 7a). However, in the presence of free nanoparticles, cell viability is greatly reduced, with high cellular death and inhibition of normal cell proliferation (bottom panel of Figure 7a). Our results thus show that, by encapsulating the nanoparticles within these silk films, mammalian cells can be shielded from the cytotoxic effects, making them more biocompatible, while also providing high antimicrobial activity.

Taken together, the biocompatibility results and the antimicrobial results strongly suggest that an optimum concentration of SF-SeNP films exists. Using a concentration of 225 μg/mL, we were able to eradicate the majority of bacteria (>70% for *E. coli* and >92% for *B. subtilis*) and fungi (>95%), while also protecting and keeping the majority of HEK-293 cells alive (>85%). This is evident in Figure 7c, which shows the graph of *B. subtilis* death (in red) and HEK-293 cell viability (in green), as a function of the silk–selenium film concentration.

## Conclusion

Antimicrobial resistance is an increasingly major healthcare problem of increasing urgency. It has become clear that alternative routes, in addition to conventional antibiotic and antifungal treatments, need to be developed. In this study, we have combined selenium nanoparticles with silk fibroin in order to form biocompatible films that exhibit strong antimicrobial action. Two different bacteria, Gram-negative *E. coli* and Gram-positive *B. subtilis* were tested, while the fungi *C. parapsilosis* was also investigated. In all cases, it was found that the films displayed potent antibacterial and antifungal properties. Furthermore, the biocomatibility of the films was investigated via an MTT-based cell viability assay. HEK-293 mammalian cell viability was monitored and it was established that in the presence of films containing low nanoparticle concentrations, high cellular viability was observed. This is primarily because the protein scaffold has the ability to shield and protect the mammalian cells from the cytotoxic effects of the bare nanoparticles. By combining the results from the antimicrobial assays and the mammalian cell viability assays, it was determined that the optimum concentration which displays both high biocompatibility and high bacterial and fungal death was 225 μg/mL. This optimum film condition had the ability to kill the majority of bacteria and fungi while also keeping the majority of HEK-293 cells alive. Since bacteria and fungi are unlikely to develop antimicrobial resistance to the nanoparticles embedded within the films, these hybrid organic/inorganic bioinspired materials provide a basis for potential biomedical and biotechnological applications, especially for use as topical antimicrobial agents.

As potential future studies, we intend to investigate more clinically relevant bacteria and fungi, such as *Staphylococcus aureus* and *Candida albicans*, respectively. Such bacteria and fungi have been known to be particularly difficult to tackle, especially in a nosocomial setting, so targeting these micro-organisms would be a major step forward.^57^ Furthermore, we intend to integrate our SeNP–silk hybrid material with more-advanced formulation systems, such as inkjet printing of polymeric materials with antimicrobial properties.^58^ Using such an approach, we would be able to tailor the geometry of the printed devices which could have further applications in a nosocomial environment.

## Supporting Information Available

Optical micrographs of aggregated selenium nanoparticles (SeNPs); TEM and EDX spectra of SeNPs; XRD spectra of SeNPs; Images of nanoparticle solutions and corresponding films following formation; Images of *E. coli* colonies on agar plates with and without silk–SeNP films; and bright-field and confocal micrographs of *E. coli* with and without the presence of silk films, stained with SYTOX blue to monitor membrane disruption (PDF)

## References

[ref1] Zhang S. Fabrication of Novel Biomaterials Through Molecular Self-Assembly. Nat. Biotechnol. 2003, 21, 1171–1178. 10.1038/nbt874. 14520402

[ref2] Hakala T. A.; Davies S.; Toprakcioglu Z.; Bernardim B.; Bernardes G. J.; Knowles T. P. A Microfluidic Co-Flow Route for Human Serum Albumin-Drug-Nanoparticle Assembly. Chem.—Eur. J. 2020, 26, 5965–5969. 10.1002/chem.202001146. 32237164 PMC7318336

[ref3] Schnaider L.; Toprakcioglu Z.; Ezra A.; Liu X.; Bychenko D.; Levin A.; Gazit E.; Knowles T. P. Biocompatible Hybrid Organic/Inorganic Microhydrogels Promote Bacterial Adherence and Eradication in Vitro and in Vivo. Nano Lett. 2020, 20, 1590–1597. 10.1021/acs.nanolett.9b04290. 32040332

[ref4] Shen Y.; Levin A.; Kamada A.; Toprakcioglu Z.; Rodriguez-Garcia M.; Xu Y.; Knowles T. P. From Protein Building Blocks to Functional Materials. ACS Nano 2021, 15, 5819–5837. 10.1021/acsnano.0c08510. 33760579 PMC8155333

[ref5] Toprakcioglu Z.; Hakala T. A.; Levin A.; Becker C. F.; Bernandes G. G.; Knowles T. P. Multi-Scale Microporous Silica Microcapsules from Gas-in Water-in Oil Emulsions. Soft Matter 2020, 16, 3082–3087. 10.1039/C9SM02274K. 32140697

[ref6] Yuan C.; Ji W.; Xing R.; Li J.; Gazit E.; Yan X. Hierarchically Oriented Organization in Supramolecular Peptide Crystals. Nat. Rev. Chem. 2019, 3, 567–588. 10.1038/s41570-019-0129-8. PMC7617026 39649433

[ref7] Adler-Abramovich L.; Gazit E. The Physical Properties of Supramolecular Peptide Assemblies: From Building Block Association to Technological Applications. Chem. Soc. Rev. 2014, 43, 6881–6893. 10.1039/C4CS00164H. 25099656

[ref8] Shimanovich U.; Michaels T. C.; De Genst E.; Matak-Vinkovic D.; Dobson C. M.; Knowles T. P. Sequential Release of Proteins from Structured Multishell Microcapsules. Biomacromolecules 2017, 18, 3052–3059. 10.1021/acs.biomac.7b00351. 28792742

[ref9] Varanko A.; Saha S.; Chilkoti A. Recent Trends in Protein and Peptide-based Biomaterials for Advanced Drug Delivery. Adv. Drug Delivery Rev. 2020, 156, 133–187. 10.1016/j.addr.2020.08.008. PMC7456198 32871201

[ref10] Liu X.; Toprakcioglu Z.; Dear A. J.; Levin A.; Ruggeri F. S.; Taylor C. G.; Hu M.; Kumita J. R.; Andreasen M.; Dobson C. M.; Shimanovich U.; Knowles T. P. Fabrication and Characterization of Reconstituted Silk Microgels for the Storage and Release of Small Molecules. Macromol. Rapid Commun. 2019, 40, 1800898 10.1002/marc.201800898. 30840348

[ref11] Khalid A.; Mitropoulos A. N.; Marelli B.; Tomljenovic-Hanic S.; Omenetto F. G. Doxorubicin Loaded Nanodiamond-Silk Spheres for Fluorescence Tracking and Controlled Drug Release. Biomedical Optics Express 2016, 7, 132 10.1364/BOE.7.000132. 26819823 PMC4722898

[ref12] Toprakcioglu Z.; Knowles T. P. Shear-mediated Sol-Gel Transition of Regenerated Silk Allows the Formation of Janus-like Microgels. Sci. Rep. 2021, 11, 10.1038/s41598-021-85199-1. PMC7988050 33758259

[ref13] Toprakcioglu Z.; Kumar Challa P.; Morse D. B.; Knowles T. Attoliter Protein Nanogels from Droplet Nanofluidics for Intracellular Delivery. Sci. Adv. 2020, 6, 10.1126/sciadv.aay7952. PMC7007244 32083185

[ref14] Xing R.; Liu K.; Jiao T.; Zhang N.; Ma K.; Zhang R.; Zou Q.; Ma G.; Yan X. An Injectable Self-Assembling Collagen-Gold Hybrid Hydrogel for Combinatorial Antitumor Photothermal/Photodynamic Therapy. Adv. Mater. 2016, 28, 3669–3676. 10.1002/adma.201600284. 26991248

[ref15] Koh L. D.; Cheng Y.; Teng C. P.; Khin Y. W.; Loh X. J.; Tee S. Y.; Low M.; Ye E.; Yu H. D.; Zhang Y. W.; Han M. Y. Structures, Mechanical Properties and Applications of Silk Fibroin Materials. Prog. Polym. Sci. 2015, 46, 86–110. 10.1016/j.progpolymsci.2015.02.001.

[ref16] Rockwood D. N.; Preda R. C.; Yücel T.; Wang X.; Lovett M. L.; Kaplan D. L. Materials Fabrication from Bombyx Mori Silk Fibroin. Nat. Protoc. 2011, 6, 1612–1631. 10.1038/nprot.2011.379. 21959241 PMC3808976

[ref17] Guo C.; Li C.; Vu H. V.; Hanna P.; Lechtig A.; Qiu Y.; Mu X.; Ling S.; Nazarian A.; Lin S. J.; Kaplan D. L. Thermoplastic Moulding of Regenerated Silk. Nat. Mater. 2020, 19, 102–108. 10.1038/s41563-019-0560-8. 31844276 PMC6986341

[ref18] Rizzo G.; Lo Presti M.; Giannini C.; Sibillano T.; Milella A.; Guidetti G.; Musio R.; Omenetto F. G.; Farinola G. M. Bombyx mori Silk Fibroin Regeneration in Solution of Lanthanide Ions: A Systematic Investigation. Front. Bioeng. Biotechnol. 2021, 9, 10.3389/fbioe.2021.653033. PMC8222627 34178956

[ref19] Vollrath F.; Porter D. Spider Silk as Archetypal Protein Elastomer. Soft Matter 2006, 2, 377–385. 10.1039/b600098n. 32680251

[ref20] Magaz A.; Roberts A. D.; Faraji S.; Nascimento T. R.; Medeiros E. S.; Zhang W.; Greenhalgh R. D.; Mautner A.; Li X.; Blaker J. J. Porous, Aligned, and Biomimetic Fibers of Regenerated Silk Fibroin Produced by Solution Blow Spinning. Biomacromolecules 2018, 19, 4542–4553. 10.1021/acs.biomac.8b01233. 30387602

[ref21] Schnaider L.; Ghosh M.; Bychenko D.; Grigoriants I.; Ya’Ari S.; Shalev Antsel T.; Matalon S.; Sarig R.; Brosh T.; Pilo R.; Gazit E.; Adler-Abramovich L. Enhanced Nanoassembly-Incorporated Antibacterial Composite Materials. ACS Appl. Mater. Interfaces 2019, 11, 21334 10.1021/acsami.9b02839. 31134790

[ref22] Zou X.; Jiang Z.; Li L.; Huang Z. Selenium Nanoparticles Coated with pH Responsive Silk Fibroin Complex for Fingolimod Release and Enhanced Targeting in Thyroid Cancer. Artif. Cells, Nanomed. Biotechnol. 2021, 49, 83–95. 10.1080/21691401.2021.1871620. 33438446

[ref23] Larsson D. G. J.; Flach C.-F. Antibiotic Resistance in the Environment. Nat. Rev. Microbiol. 2022, 20, 257 10.1038/s41579-021-00649-x. 34737424 PMC8567979

[ref24] Prestinaci F.; Pezzotti P.; Pantosti A. Antimicrobial Resistance: A Global Multifaceted Phenomenon. Pathogens Global Health 2015, 109, 309–318. 10.1179/2047773215Y.0000000030. 26343252 PMC4768623

[ref25] Debroas D.; Siguret C. Viruses as Key Reservoirs of Antibiotic Resistance Genes in the Environment. ISME J. 2019, 13, 2856–2867. 10.1038/s41396-019-0478-9. 31358910 PMC6794266

[ref26] AshaRani P. V.; Mun G. L. K.; Hande M. P.; Valiyaveettil S. Cytotoxicity and Genotoxicity of Silver Nanoparticles in Human Cells. ACS Nano 2009, 3, 279–290. 10.1021/nn800596w. 19236062

[ref27] Le Ouay B.; Stellacci F. Antibacterial Activity of Silver Nanoparticles: A Surface Science Insight. Nano Today 2015, 10, 339–354. 10.1016/j.nantod.2015.04.002.

[ref28] Salas-Orozco M.; Niño-Martínez N.; Martínez-Castañón G.-A.; Méndez F. T.; Jasso M. E. C.; Ruiz F. Mechanisms of Resistance to Silver Nanoparticles in Endodontic Bacteria: A Literature Review. J. Nanomater. 2019, 2019, 1 10.1155/2019/7630316.

[ref29] Rayman M. P. Selenium and Human Health. Lancet 2012, 379, 1256–68. 10.1016/S0140-6736(11)61452-9. 22381456

[ref30] Karnaukh E. A.; Walker L. M.; Lynch K. A.; Wiita E. G.; Buzzeo M. C. Electrochemical Study of Selenocystine Reactivity and Reduction at Metallic Surfaces. ChemElectroChem. 2017, 4, 1250–1255. 10.1002/celc.201600717.

[ref31] Evans S. O.; Khairuddin P. F.; Jameson M. B. Optimising Selenium for Modulation of Cancer Treatments. Anticancer Res. 2017, 37, 6497–6509. 10.21873/anticanres.12106. 29187424

[ref32] Filipović N.; Ušjak D.; Milenković M. T.; Zheng K.; Liverani L.; Boccaccini A. R.; Stevanović M. M. Comparative Study of the Antimicrobial Activity of Selenium Nanoparticles With Different Surface Chemistry and Structure. Front. Bioeng. Biotechnol. 2021, 8, 10.3389/fbioe.2020.624621. PMC7869925 33569376

[ref33] Kuršvietienė L.; Mongirdienė A.; Bernatonienė J.; Šulinskienė J.; Stanevičienė I. Selenium Anticancer Properties and Impact on Cellular Redox Status. Antioxidants 2020, 9, 80 10.3390/antiox9010080. 31963404 PMC7023255

[ref34] Chen W.; Li Y.; Yang S.; Yue L.; Jiang Q.; Xia W. Synthesis and Antioxidant Properties of Chitosan and Carboxymethyl Chitosan-Stabilized Selenium Nanoparticles. Carbohydr. Polym. 2015, 132, 574–581. 10.1016/j.carbpol.2015.06.064. 26256384

[ref35] Zou J.; Su S.; Chen Z.; Liang F.; Zeng Y.; Cen W.; Zhang X.; Xia Y.; Huang D. Hyaluronic Acid-Modified Selenium Nanoparticles for Enhancing the Therapeutic Efficacy of Paclitaxel in Lung Cancer Therapy. Artificial Cells, Nanomed. Biotechnol. 2019, 47, 3456–3464. 10.1080/21691401.2019.1626863. 31469318

[ref36] Mary T. A.; Shanthi K.; Vimala K.; Soundarapandian K. PEG Functionalized Selenium Nanoparticles as a Carrier of Crocin to Achieve Anticancer Synergism. RSC Adv. 2016, 6, 22936–22949. 10.1039/C5RA25109E.

[ref37] Qiu W. Y.; Wang Y. Y.; Wang M.; Yan J. K. Construction, Stability, and Enhanced Antioxidant Activity of Pectin-Decorated Selenium Nanoparticles. Colloids Surf., B 2018, 170, 692–700. 10.1016/j.colsurfb.2018.07.003. 29986266

[ref38] Pi J.; Jiang J.; Cai H.; Yang F.; Jin H.; Yang P.; Cai J.; Chen Z. W. Ge11 Peptide Conjugated Selenium Nanoparticles for egfr Targeted Oridonin Delivery to Achieve Enhanced Anticancer Efficacy by Inhibiting egfr-Mediated pi3k/akt and ras/raf/mek/erk Pathways. Drug Delivery 2017, 24, 1549–1564. 10.1080/10717544.2017.1386729. 29019267 PMC6920706

[ref39] Ferro C.; Florindo H. F.; Santos H. A. Selenium Nanoparticles for Biomedical Applications: From Development and Characterization to Therapeutics. Adv. Healthcare Mater. 2021, 10, 2100598 10.1002/adhm.202100598. 34121366

[ref40] Geoffrion L. D.; Hesabizadeh T.; Medina-Cruz D.; Kusper M.; Taylor P.; Vernet-Crua A.; Chen J.; Ajo A.; Webster T. J.; Guisbiers G. Naked Selenium Nanoparticles for Antibacterial and Anticancer Treatments. ACS Omega 2020, 5, 2660 10.1021/acsomega.9b03172. 32095689 PMC7033664

[ref41] Guisbiers G.; Wang Q.; Khachatryan E.; Mimun L. C.; Mendoza-Cruz R.; Larese-Casanova P.; Webster T. J.; Nash K. L. Inhibition of E. coli and S. aureus with Selenium Nanoparticles Synthesized by Pulsed Laser Ablation in Deionized Water. Int. J. Nanomed. 2016, 11, 3731–3736. 10.2147/IJN.S106289. PMC4982524 27563240

[ref42] Lara H. H.; Guisbiers G.; Mendoza J.; Mimun L. C.; Vincent B. A.; Lopez-Ribot J. L.; Nash K. L. Synergistic Antifungal Effect of Chitosan-Stabilized Selenium Nanoparticles Synthesized by Pulsed Laser Ablation in Liquids Against *Candida Albicans* Biofilms. Int. J. Nanomed. 2018, 13, 2697–2708. 10.2147/IJN.S151285. PMC5937483 29760550

[ref43] Vahdati M.; Tohidi Moghadam T. Synthesis and Characterization of Selenium Nanoparticles-Lysozyme Nanohybrid System with Synergistic Antibacterial Properties. Sci. Rep. 2020, 10, 10.1038/s41598-019-57333-7. PMC6965607 31949299

[ref44] Cruz L. Y.; Wang D.; Liu J. Biosynthesis of Selenium Nanoparticles, Characterization and X-ray Induced Radiotherapy for the Treatment of Lung Cancer with Interstitial Lung Disease. J. Photochem. Photobiol. B: Biol. 2019, 191, 123–127. 10.1016/j.jphotobiol.2018.12.008. 30616036

[ref45] Bini E.; Knight D. P.; Kaplan D. L. Mapping Domain Structures in Silks from Insects and Spiders Related to Protein Assembly. J. Mol. Biol. 2004, 335, 27–40. 10.1016/j.jmb.2003.10.043. 14659737

[ref46] Zhou C.-Z.; Confalonieri F.; Medina N.; Zivanovic Y.; Esnault C.; Yang T.; Jacquet M.; Janin J.; Duguet M.; Perasso R.; Li Z.-G. Fine Organization of Bombyx mori Fibroin Heavy Chain Gene. Nucleic Acids Res. 2000, 28, 2413–2419. 10.1093/nar/28.12.2413. 10871375 PMC102737

[ref47] Arakha M.; Pal S.; Samantarrai D.; Panigrahi T. K.; Mallick B. C.; Pramanik K.; Mallick B.; Jha S. Antimicrobial Activity of Iron Oxide Nanoparticle Upon Modulation of Nanoparticle-Bacteria Interface. Sci. Rep. 2015, 5, 10.1038/srep14813. PMC4594095 26437582

[ref48] Slavin Y. N.; Asnis J.; Häfeli U. O.; Bach H. Metal Nanoparticles: Understanding the Mechanisms Behind Antibacterial Activity. J. Nanobiotechnol. 2017, 15, 10.1186/s12951-017-0308-z. PMC5627441 28974225

[ref49] Makabenta J. M. V.; Nabawy A.; Li C. H.; Schmidt-Malan S.; Patel R.; Rotello V. M. Nanomaterial-Based Therapeutics for Antibiotic-Resistant Bacterial Infections. Nat. Rev. Microbiol. 2021, 19, 23–36. 10.1038/s41579-020-0420-1. 32814862 PMC8559572

[ref50] Zhao G.; Wu X.; Chen P.; Zhang L.; Yang C. S.; Zhang J. Selenium Nanoparticles are More Efficient than Sodium Selenite in Producing Reactive Oxygen Species and Hyper-Accumulation of Selenium Nanoparticles in Cancer Cells Generates Potent Therapeutic Effects. Free Radical Biol. Med. 2018, 126, 55–66. 10.1016/j.freeradbiomed.2018.07.017. 30056082

[ref51] Zhuang Y.; Li L.; Feng L.; Wang S.; Su H.; Liu H.; Liu H.; Wu Y. Mitochondrion-Targeted Selenium Nanoparticles Enhance Reactive Oxygen Species-Mediated Cell Death. Nanoscale 2020, 12, 1389–1396. 10.1039/C9NR09039H. 31913383

[ref52] Stoimenov P. K.; Klinger R. L.; Marchin G. L.; Klabunde K. J. Metal Oxide Nanoparticles as Bactericidal Agents. Langmuir 2002, 18, 6679–6686. 10.1021/la0202374.

[ref53] Ramalingam B.; Parandhaman T.; Das S. K. Antibacterial Effects of Biosynthesized Silver Nanoparticles on Surface Ultrastructure and Nanomechanical Properties of Gram-Negative Bacteria viz. *Escherichia coli* and *Pseudomonas aeruginosa*. ACS Appl. Mater. Interfaces 2016, 8, 4963–4976. 10.1021/acsami.6b00161. 26829373

[ref54] Gupta A.; Mumtaz S.; Li C. H.; Hussain I.; Rotello V. M. Combatting Antibiotic-Resistant Bacteria Using Nanomaterials. Chem. Soc. Rev. 2019, 48, 415–427. 10.1039/C7CS00748E. 30462112 PMC6340759

[ref55] Fayaz A. M.; Balaji K.; Girilal M.; Yadav R.; Kalaichelvan P. T.; Venketesan R. Biogenic Synthesis of Silver Nanoparticles and their Synergistic Effect with Antibiotics: A Study Against Gram-positive and Gram-negative Bacteria. Nanomed.: Nanotechnol., Biol., Med. 2010, 6, 103–109. 10.1016/j.nano.2009.04.006. 19447203

[ref56] Lee N. Y.; Ko W. C.; Hsueh P. R. Nanoparticles in the Treatment of Infections Caused by Multidrug-Resistant Organisms. Front. Pharmacol. 2019, 10, 10.3389/fphar.2019.01153. PMC6787836 31636564

[ref57] Peleg A. Y.; Hogan D. A.; Mylonakis E. Medically Important Bacterial-Fungal Interactions. Nat. Rev. Microbiol. 2010, 8, 340–349. 10.1038/nrmicro2313. 20348933

[ref58] Wales D. J.; Miralles-Comins S.; Franco-Castillo I.; Cameron J. M.; Cao Q.; Karjalainen E.; Alves Fernandes J.; Newton G. N.; Mitchell S. G.; Sans V. Decoupling Manufacturing from Application in Additive Manufactured Antimicrobial Materials. Biomater. Sci. 2021, 9, 5397 10.1039/D1BM00430A. 33988192

