# Supplementary material for: Selenium Silk Nanostructured Films with Antifungal and Antibacterial Activity
Source: ACS Appl Mater Interfaces. 2023 Feb 20;15(8):10452–63. doi: 10.1021/acsami.2c21013 (PMC9982822; doi:10.1021/acsami.2c21013)
Supplement: Supplementary file 1 — am2c21013_si_001.pdf [file am2c21013_si_001.pdf]

Selenium Silk Nanostructured Films with Antifungal and Antibacterial Activity

Zenon Toprakcioglu,[†,‡] Elizabeth G. Wiita,[†,‡] Akhila K. Jayaram,[†,¶,‡] Rebecca C. Gregory,[†] and Tuomas P. J. Knowles[*,†,¶]

†*Yusuf Hamied Department of Chemistry, University of Cambridge, Lensfield Road, Cambridge CB2 1EW, UK*

‡*These authors contributed equally*

¶*Cavendish Laboratory, Department of Physics, University of Cambridge, J J Thomson Avenue, Cambridge CB3 0HE, UK*

E-mail: tpjk2@cam.ac.uk

Supporting Information

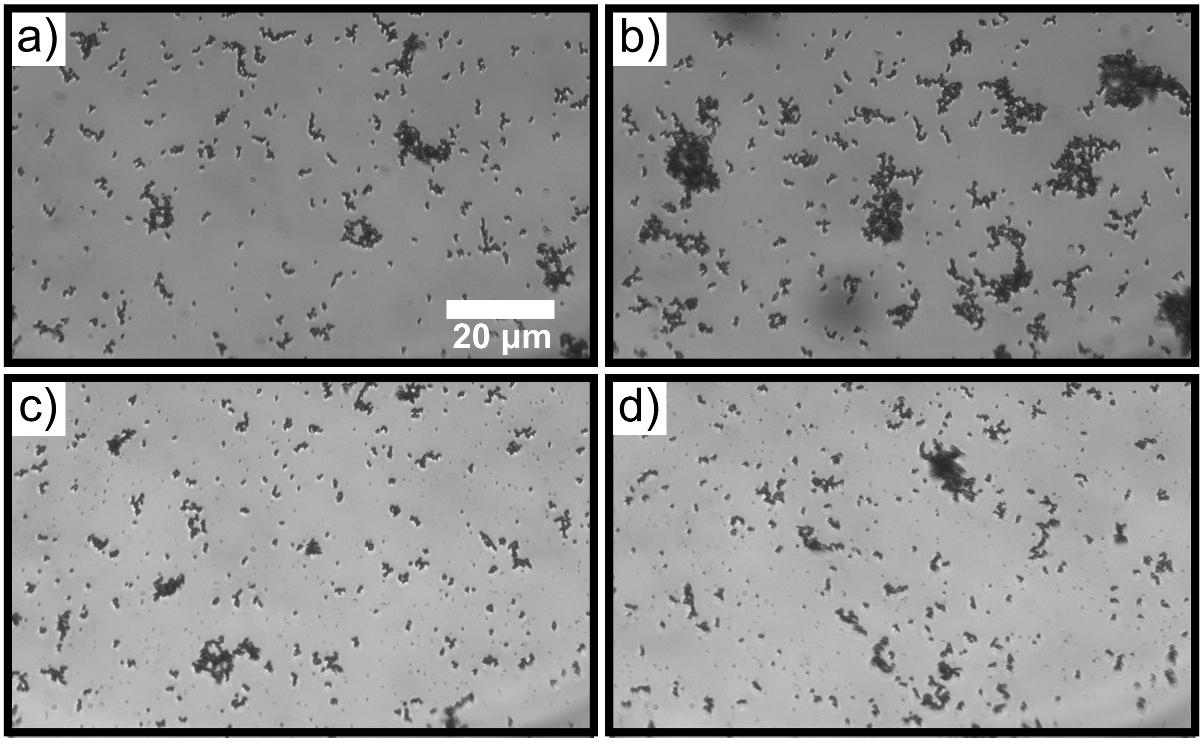

Figure S1: (a-d) Se nanoparticle agglomeration monitored over time. After day 6 of formation, it was observed that the nanoparticles tend to clump into micrometer-sized structures.

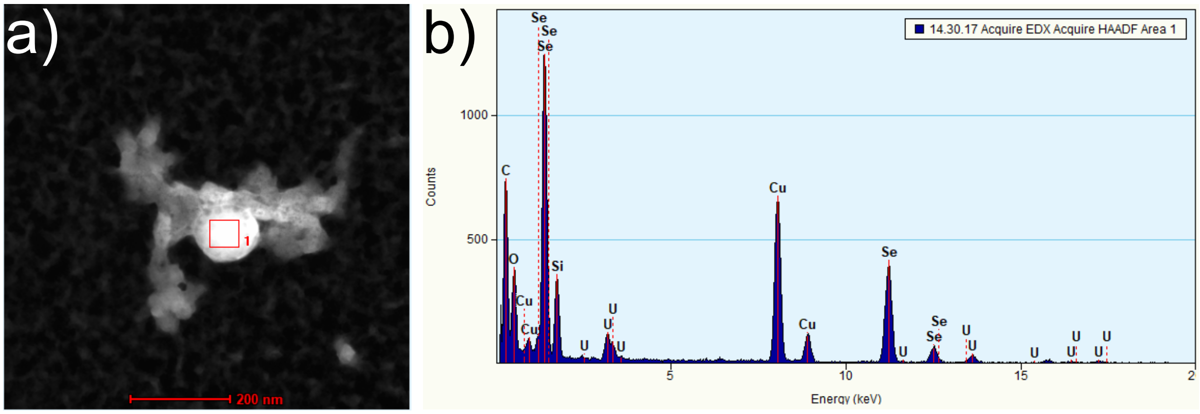

Figure S2: **(a)** TEM micrograph of nanoparticles formed using a 600 μg/mL sodium selenite solution. **(b)** EDX spectrum of the corresponding nanoparticle solution

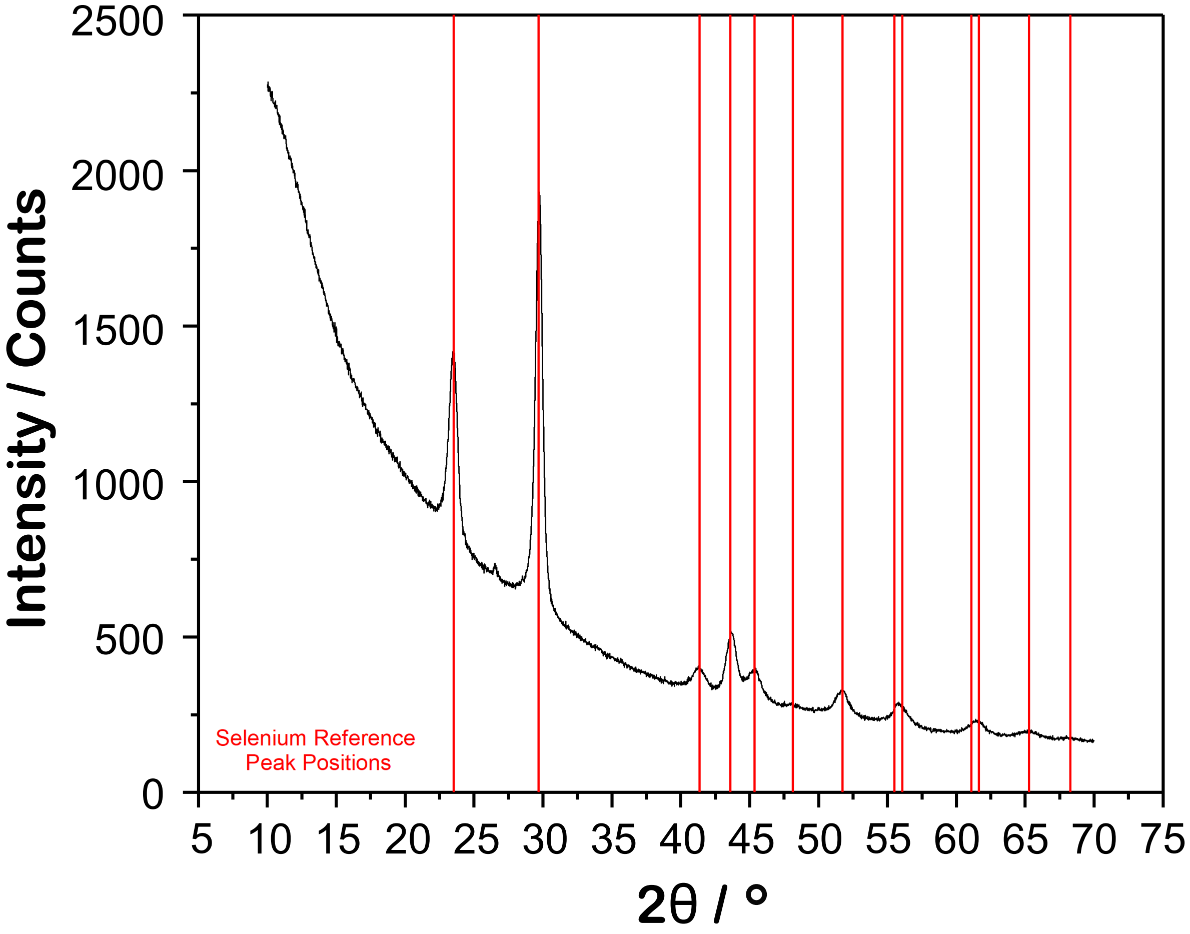

Figure S3: XRD spectrum of a 600 μg/mL selenium nanoparticle solution.

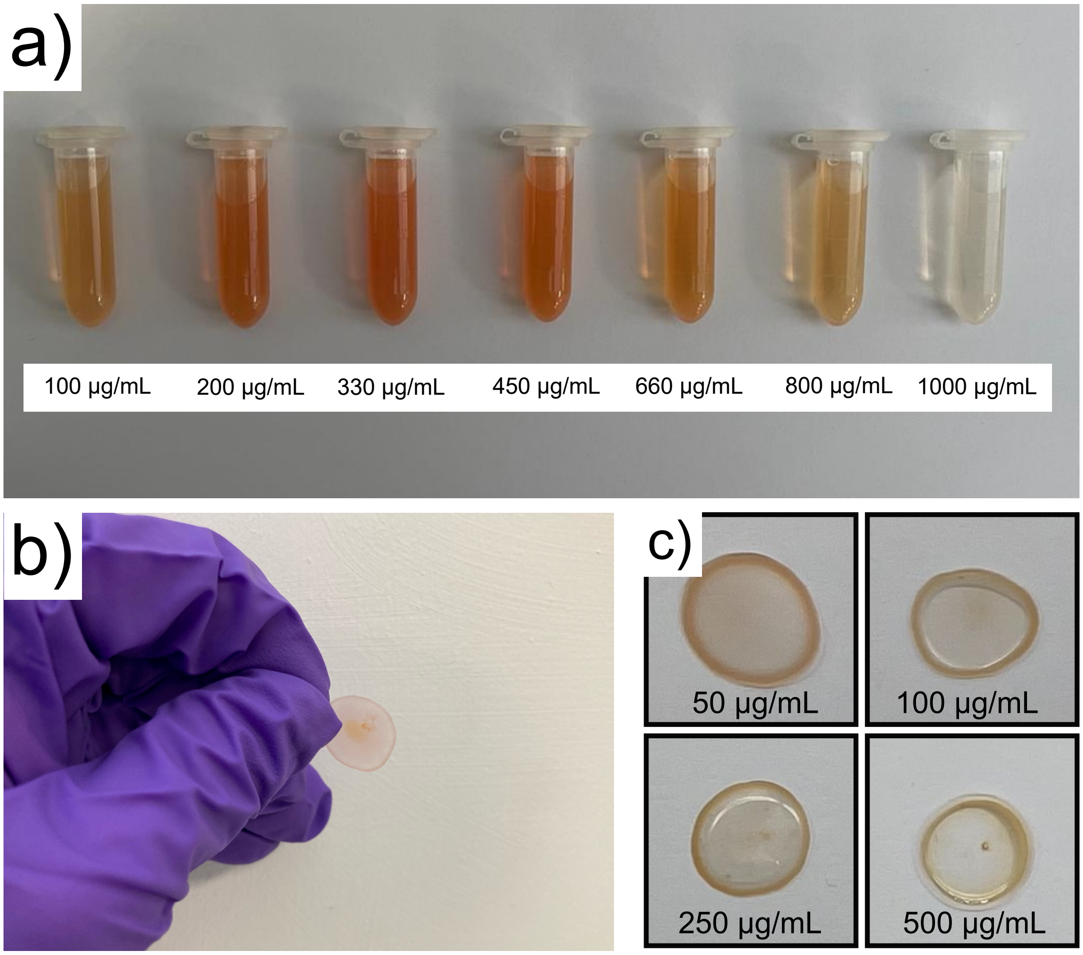

Figure S4: (**a**) Image of the 7 SeNP systems tested following 1 day of incubation. (**b-c**) Images of films made using the hybrid inorganic/organic silk SeNP system.

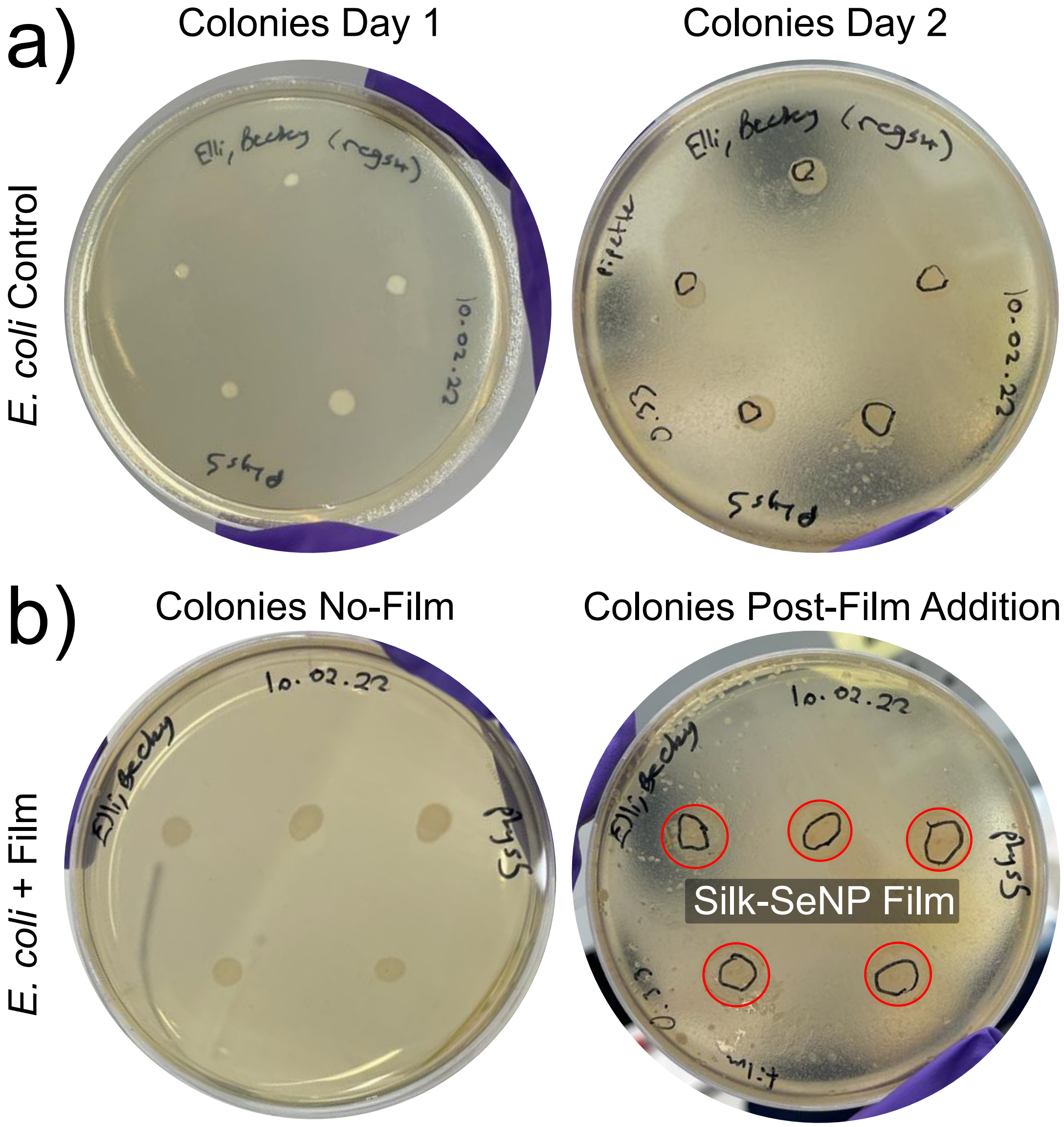

Figure S5: (**a**) Images of *E. coli* colonies on agar plates in the absence of silk-SeNP films. (**b**) Images of *E. coli* colonies on agar plates in the presence of silk-SeNP films. The black lines outline the bacterial colonies on Day 1, whereas the red lines outline the addition of the antimicrobial films.

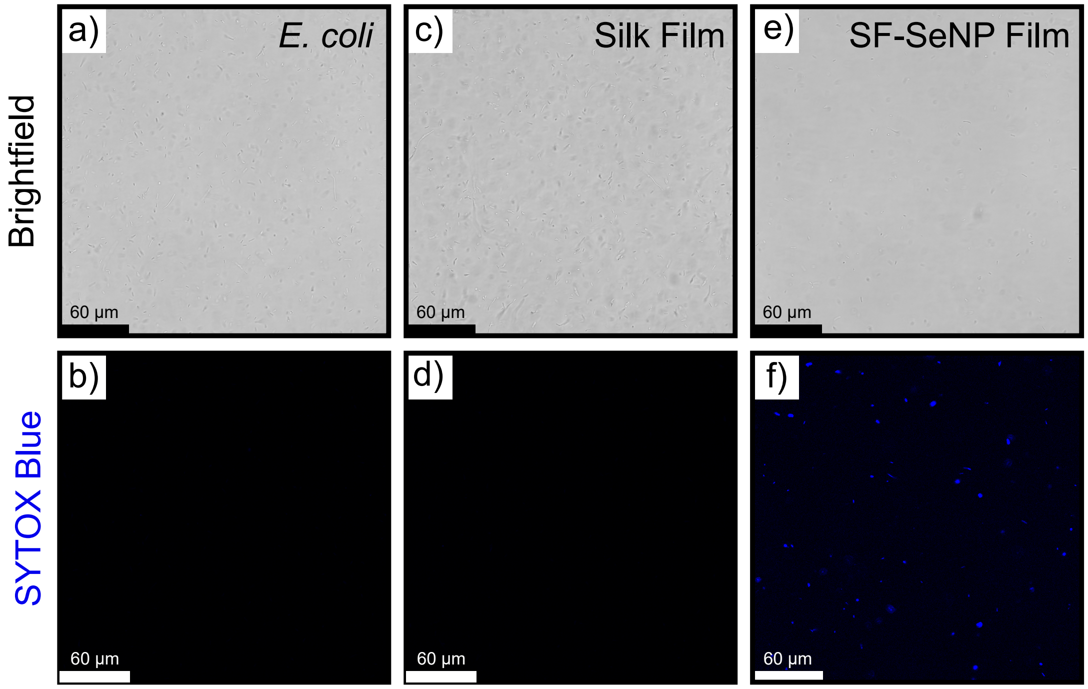

Figure S6: (**a-f**) Brightfield and confocal micrographs of *E. coli* with and without the presence of silk films. (**a-b**) *E. coli* control. (**c-d**) *E. coli* incubated with silk films. Brightfield images indicate that bacteria proliferated on the film while no SYTOX Blue staining of cell membranes was observed (**e-f**) *E. coli* incubated with SF-SeNP Films. Brightfield images show a decreased number of bacteria. SYTOX Blue staining of bacterial membranes was observed, indicating membrane disruption.
